# Adaptive Space-Aware Infotaxis II as a Strategy for Odor Source Localization

**DOI:** 10.3390/e26040302

**Published:** 2024-03-29

**Authors:** Shiqi Liu, Yan Zhang, Shurui Fan

**Affiliations:** 1 Innovation and Research Institute, Hebei University of Technology, Shijiazhuang 050299, China; 202121902015@stu.hebut.edu.cn (S.L.); fansr@hebut.edu.cn (S.F.); 2 School of Electronic and Information Engineering, Hebei University of Technology, Tianjin 300401, China

**Keywords:** odor source localization, information entropy, Bayesian inference, adaptive navigation, salp swarm algorithm

## Abstract

Mobile robot olfaction of toxic and hazardous odor sources is of great significance in anti-terrorism, disaster prevention, and control scenarios. Aiming at the problems of low search efficiency and easily falling into a local optimum of the current odor source localization strategies, the paper proposes the adaptive space-aware Infotaxis II algorithm. To improve the tracking efficiency of robots, a new reward function is designed by considering the space information and emphasizing the exploration behavior of robots. Considering the enhancement in exploratory behavior, an adaptive navigation-updated mechanism is proposed to adjust the movement range of robots in real time through information entropy to avoid an excessive exploration behavior during the search process, which may lead the robot to fall into a local optimum. Subsequently, an improved adaptive cosine salp swarm algorithm is applied to confirm the optimal information adaptive parameter. Comparative simulation experiments between ASAInfotaxis II and the classical search strategies are carried out in 2D and 3D scenarios regarding the search efficiency and search behavior, which show that ASAInfotaxis II is competent to improve the search efficiency to a larger extent and achieves a better balance between exploration and exploitation behaviors.

## 1. Introduction

Odor source localization (OSL) is important for anti-terrorist attacks and disaster emergency response, such as the leakage of toxic flammable and explosive gases [1], nuclear accidents [2], volcanic eruptions [3], as well as multiple scenarios. The occurrence of these events has a considerable impact on human safety, atmospheric environment, and many other aspects. The release of these odor sources may pose a threat to human health, or perhaps provide clues to the location of a resource. The ability to identify and quantify these sources, to monitor source emissions, or to respond quickly to an incident is critical.

Early odor source localization is usually processed using static sensor networks and weather station networks [4,5], through which the early detection of places of interest or strategically important sites can be performed. However, static sensing network methods have significantly long sampling times, limited measurement ranges, and relatively high installation and maintenance costs. Therefore, with the rapid development of small sensors and smart mobile search devices [6], which are competent to overcome the problems of maintenance, power supply, network design, and cost of large static sensor networks [7], searching for release sources using smart mobile search devices is gradually becoming a mainstream approach to source localization. At present, mobile robot olfaction is mainly divided into three types of situations: reactive strategies, heuristic strategies, and cognitive strategies.

Reactive strategies are the earliest situations in the field of OSL performed by mobile robots, and draw inspiration from the behavior of organisms searching for food, mates, or other predators, e.g., moths [8] and Escherichia coli [9], for the purpose of autonomously tracking chemical plumes. Reactive strategies are generally gradient-based, e.g., zigzag–silkworm [10] and spiral–surge [11], in which the robot moves repeatedly along the chemical concentration gradient, constantly steering so that the robot moves to the side with a higher chemical concentration. Reactive strategies are suitable for diffusion-dominated airflow environments and are usually used for micro-vehicles or ground robots to search for odor sources in indoor environments without a strong airflow [12]. After that, some researchers have performed OSL by some heuristic strategies, which are generally designed for multiple robots and consider OSL as a mathematical optimization problem. During the optimization process, different optimization algorithms can be chosen to update the objective function, such as whale optimization algorithm [13], particle swarm optimization [14], and reinforcement learning methods [15]. However, most real-world scenarios are dominated by turbulence, and the fluctuations generated by turbulence make the search process more complicated. The instantaneous concentration of odors presents a curved and intermittent structure, at which time there is no more accurate concentration gradient, and thus the robot is prone to falling into a local optimum during the OSL process, leading to the failure of reactive search strategies as well as heuristic strategies. Cognitive search strategies based on probabilistic inference are built on the foundation of Bayesian theory, which provides a statistically rigorous approach to deal with uncertainty in the inference process [16]. A variety of stochastic factors, such as sensor errors and intermittent turbulence, can be taken into account while incorporating the available observational data, which provide stronger robustness under the condition of sparse cues. Cassandra et al. [17] investigated the uncertainty of mobile robot navigation through discrete Bayesian models. Later, Vergassola et al. [18] introduced information entropy based on the Bayesian model and proposed Infotaxis. Infotaxis has now been successfully applied to many OSL tasks and demonstrated a fast and stable search capability [19]. Ristic et al. [20] implemented autonomous search for release sources in unknown environments using Rao-Blackwellised particle filters with entropy-decreasing motion control in a Bayesian framework. Eggels et al. [21] implemented Infotaxis in three dimensions. Ruddick et al. [22] evaluated the improved performance of Infotaxis under different environmental conditions, which showed that Infotaxis had an excellent OSL performance at high wind speeds.

In recent years, new cognitive search strategies have been proposed. Hutchinson et al. [23] designed a reward function based on maximum entropy sampling, known as the Entrotaxis, but the function performs poorly in two dimensions, which may be caused by the strong gradient near the source in the model, leading to biased decisions based on local random samples. Song et al. [24] proposed a reward function that combines entropy and potential energy, which explores more information through entropy and uses the potential energy to enhance the chasing behavior as uncertainty recedes, effectively solving the problem of the classic Infotaxis being more likely to fall into local self-trapping. Rahbar et al. [25] designed an enhanced navigation strategy that allowed the robot to maintain a balance between exploration and exploitation behaviors during the search process and used the Metropolis–Hasting method [26] for the estimation of the source location parameters, which reduces the cost associated with the probabilistic inference computation. Ji et al. [27] proposed the MEGI-taxis, which reconstructs the posterior probability density function (PDF) using a Gaussian mixture model (GMM) and guides the searcher by using the maximum effective Gaussian distribution (MEGI), performing more accurately and quickly than other cognitive strategies in turbulent environments. Park et al. [28] combined Infotaxis with the GMM to propose GMM-Infotaxis, where the GMM appropriately clustered all possible source locations to promote more exploitation behaviors, which contributed to a better trade-off between exploration and exploitation. Zhao et al. [29] designed a passive evasion mechanism for the problem of source searching in randomly obstructed environments by marking forbidden zones and allowing the searcher to be forced out of the cluttered location to improve the performance of the cognitive strategy in obstructed environments. To address the real-life problem of obstacles such as buildings obstructing the search path and interfering with the flow of air sources, An et al. [30] introduced rapidly exploring random trees (RRTs) as a local path planner for source search to generate efficient paths in a continuous domain filled with obstacles, and RRT-Infotaxis can autonomously find a balance between exploration and exploitation.

Although the above literature improved Infotaxis to a certain extent, it still suffers from the problems of a lower search efficiency and the failure to obtain a better balance between exploration and exploitation behaviors, which leads the robot to fall into a local optimum during the search process. In this regard, this paper proposes the adaptive space-aware Infotaxis II (ASAInfotaxis II). Aiming at the problem of the low efficiency of the current cognitive strategies, a reward function focusing on the exploration behavior is designed. From the idea of balancing the exploration and exploitation behaviors, an adaptive navigation-updated mechanism is proposed, which adjusts the moving range of robots in each step in real time through information entropy to prevent the robot from falling into the local optimum in the process. For different application scenarios, the adaptive cosine salp swarm algorithm (ACSSA) is used to find the optimal information adaptive parameter to obtain the optimal search path.

The remainder of this paper is organized as follows. Section 2 describes the mathematical models for the plume model, gas sensing model, and robotic cognitive search strategies. Section 3 provides the conceptual solution of ASAInfotaxis II, including the design of a reward function, the adaptive navigation-updated mechanism, and the adaptive cosine salp swarm algorithm. Section 4 conducts numerical simulation experiments to compare the differences in search performance and search behavior between ASAInfotaxis II and several classical cognitive search strategies. In the last section, the conclusions and future work are presented.

## 2. Materials

### 2.1. Isometric Plume Model

The airflow in a macroscopic scene is almost always in a turbulent state. After being subjected to turbulence, the plume is twisted into various kinds of jagged fragment-like morphologies, and the position of the plume changes constantly with the wind speed and direction, which make it difficult to express the motion state of the plume. In this paper, an isotropic plume model, which is commonly used in turbulent environments, was used to characterize the random distribution and sparseness of plumes. In this model, detectable “particles” are emitted by the odor source at a rate of Q, and the emitted particles have a finite lifetime τ, are transported by a mean wind V, and propagate with isotropous effective diffusivity *D* [31,32]. Assuming that the wind is blowing in the negative direction along the x-axis, the average stationary concentration field $C\left(r|r_{0}\right)$ produced by the odor source located at $r_{0}$ satisfies the following advection–diffusion equation [33]:

(1) $$0=V\nabla_{x}C\left(r|r_{0}\right)+D∆C\left(r|r_{0}\right)-\frac{1}{\tau }C\left(r|r_{0}\right)+R\delta (r-r_{0})$$

Then, in 2D, the analytical solution of the concentration field $C\left(r|r_{0}\right)$ is:

(2) $$C\left(r|r_{0}\right)=\frac{Q}{2\pi D}e^{\frac{\left(x_{0}-x\right)V}{2D}}K_{0}\left(\frac{\left|r-r_{0}\right|}{\lambda }\right)$$

where $\lambda =\sqrt{\left(D\tau )/(1+\frac{V^{2}\tau }{4D}\right)}$ and $K_{0}$ denotes the zero-order corrected Bessel function. Similarly, the analytical solution of the concentration field $C\left(r|r_{0}\right)$ in 3D is:

(3) $$C\left(r|r_{0}\right)=\frac{Q}{4\pi D|r-r_{0}|}e^{\frac{\left(x_{0}-x\right)V}{2D}}e^{-\frac{\left|r-r_{0}\right|}{\lambda }}$$

According to Smoluchowski’s expression [34], a spherical object of linear size a moving into the media undergoes a series of collisions at the rate $R\left(r|r_{0}\right)$. Therefore, the model takes the capture plume cues as particle collisions and describes the spatial distribution of the cues by particle collision probability. The cue capture rate $R\left(r|r_{0}\right)$ of the robot at position r for the odor source located at $r_{0}=(x_{0},y_{0})$ is [18]:

(4) $$R\left(r|r_{0}\right)=\frac{Q}{In\left(\frac{\lambda }{a}\right)}e^{\frac{\left(x_{0}-x\right)V}{2D}}K_{0}\left(\frac{\left|r-r_{0}\right|}{\lambda }\right)$$

Similarly, the cue capture rate $R(r|r_{0})$ at position r for the odor source $r_{0}=(x_{0},y_{0},z_{0})$ in 3D is [18]:

(5) $$R\left(r|r_{0}\right)=\frac{Q}{4\pi D\left|r-r_{0}\right|}e^{\frac{\left(x_{0}-x\right)V}{2D}}e^{-\frac{\left|r-r_{0}\right|}{\lambda }}$$

### 2.2. Gas Sensing Model

When the sensor measures chemical substances diffused in a fluid, the instantaneous concentration gradient detected fluctuates greatly and is prone to sudden changes. In this paper, a binary detection sensor was utilized to process the sampled concentration, and the robot was considered to have captured the plume information when the detected value of the sensor was greater than a set threshold th, at which time the sensing result was “1”; otherwise, the sensing result was “0”. Therefore, the robot’s cue capture rate at position r is approximated as a Poisson process. The probability that the robot captures a cue at position r for k times during the time interval ∆t is [24]:

(6) $$p\left(k,r\right)=\frac{{\left[R\left(r|r_{0}\right)∆t\right]}^{k}}{k!}exp[-R\left(r|r_{0}\right)∆t]$$

### 2.3. Cognitive Search Strategy

Cognitive strategies divide the entire search area into a grid, and the robot continuously updates the PDF of the odor source location through real-time measurements from sensors. The darker the grid color, the higher the probability that the odor source is in that grid. In the initial stage of the algorithm, the whole environment map is unknown, and the probability that each grid is an odor source is equal, and the probability exhibits a uniform distribution. After each measurement, the PDF is updated according to the plume model, and the robot moves to the next target point based on the reward function. As the entropy of the PDF becomes smaller and smaller, the robot gradually approaches the odor source. The entire cognitive search process is often formulated as partially observable Markov decision processes (POMDPs) [35,36]. POMDPs consist of three main elements: an information state, a reward function, and a set of admissible actions [37]. When the PDF acts as the information state, the current knowledge about the odor source is completely specified by the PDF. The reward function maps each acceptable action as a non-negative real number that represents a measure of the expected knowledge gain, and the optimal strategy represents the action with the highest reward function. The set of allowed actions represents the set of optional positions for next step of the robot.

#### 2.3.1. Information State

As the search proceeds, the robot obtains a series of detection sequences $T_{t}=$ [$r_{t_{1}},r_{t_{2}},\cdots ,r_{t_{i}}$], which can be obtained from the trajectory $T_{t}$ by Bayesian inference with the PDF $P_{t}(r_{0})$ of the source location $r_{0}$. The trajectory $T_{t}$ is defined as:

(7) $$L_{r_{0}}\left(T_{t}\right)=\exp\left[-\int_{0}^{t}R\left(r_{t^{\prime }}|r_{0}\right)dt^{\prime }\right]\prod_{i=1}^{I}R(r_{t_{i}}|r_{0})$$

where I denotes the total number of robot captures cues along the trajectory $T_{t}$, $t_{i}$ corresponds to the moment of cue capture, $exp[-\int_{0}^{t}R(r_{t^{\prime }}|r_{0})dt^{\prime }]$ denotes that no plume cue was captured, and $\prod_{i=1}^{I}R(r_{t_{i}}|r_{0})$ denotes that a plume cue was captured.

Based on this, the $P_{t}(r_{0})$ of the odor source after the robot undertakes trajectory $T_{t}$ is:

(8) $$P_{t}(r_{0})=\frac{L_{r_{0}}\left(T_{t}\right)}{\int L_{x}\left(T_{t}\right)dr_{x}}=\frac{exp[-\int_{0}^{t}R(r_{t^{\prime }}|r_{0})dt^{\prime }]\prod_{i=1}^{I}R(r_{t_{i}}|r_{0})}{\int exp[-\int_{0}^{t}R(r_{t^{\prime }}|r_{0})dt^{\prime }]\prod_{i=1}^{I}R(r_{t_{i}}|r_{x})dr_{x}}$$

#### 2.3.2. Reward Function

Assuming that the search region is Ω, the information entropy is calculated as:

(9) $$S=-\sum_{r_{0}\in \Omega }P_{t}(r_{0})logP_{t}(r_{0})$$

The robot arrives at position r at time t, and its collected information is in $P_{t}(r_{0})$, at which point the information entropy is S. The decrease in information entropy when the robot moves to a neighboring position $r_{j}$ or remains stationary is [18]:

(10) $$\begin{array}{cc} \Delta S_{1}\left(r\to r_{j}\right)= & P_{t}\left(r_{j}\right)\left[-S\right]+\left[1-P_{t}\left(r_{j}\right)\right][p\left(0,r_{j}\right)∆S_{0}+p\left(1,r_{j}\right)∆S_{1}+\cdots \\ & \;\;+p\left({k,r}_{j}\right)∆S_{k}] \end{array}$$

where $p\left({k,r}_{j}\right)=\frac{{\left[R\left(r_{j}|r_{0}\right)∆t\right]}^{k}}{k!}exp[-R\left(r_{j}|r_{0}\right)∆t]$ and $p\left({k,r}_{j}\right)$ represents the probability that the robot captures the plume cue at the *k*th detection. $P_{t}\left(r_{j}\right)$ indicates the probability that the odor source is located at $r_{j}$. The reward function consists of two terms. The first term on the right is the exploitation term, which refers to the use of already acquired knowledge to travel to the most probable source location, and the second term on the right is the exploration term, which refers to the gathering of more information and obtaining a more reliable estimate of the odor source.

In addition, new reward functions for improving exploration and exploitation equilibrium are proposed:

The Infotaxis II [19] reward function is defined as:

(11) $$\Delta S_{2}\left(r\to r_{j}\right)=\left[1-P_{t}\left(r_{j}\right)\right][p\left(0,r_{j}\right)∆S_{0}+p\left(1,r_{j}\right)∆S_{1}+\cdots +p\left({k,r}_{j}\right)∆S_{k}]$$

The Entrotaxis [23] reward function is defined as:

(12) $$\Delta S_{3}\left(r\to r_{j}\right)=-\int P_{t}\left(r_{j}\right)logP_{t}\left(r_{j}\right)dr_{j}$$

The Sinfotaxis [38] reward function is defined as:

(13) $$\begin{array}{cc} \Delta S_{4}\left(r\to r_{j}\right)= & (\sum_{\left|\left|r-r_{0}\right|\right|\leq L_{th}}P_{t}\left(r_{j}\right))\left[-S\right]\\ & \;\;+\left(\sum_{\left|\left|r-r_{0}\right|\right|>L_{th}}P_{t}\left(r_{j}\right)\right)[p\left({0,r}_{j}\right)∆S_{0}+p\left({1,r}_{j}\right)∆S_{1}+\cdots \\ & \;\;+p\left({k,r}_{j}\right)∆S_{k}] \end{array}$$

where $L_{th}$ is the threshold value that considers the distance to find the odor source.

#### 2.3.3. A Set of Admissible Actions

**Figure 1:**
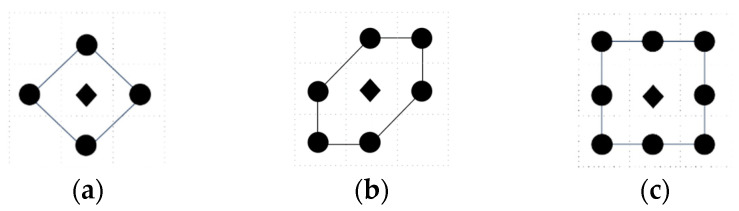
Schematic diagram of admissible action sets in a 2D space: (**a**) 4-direction; (**b**) 6-direction; and (**c**) 8-direction sets.

In 2D, a set of admissible actions is available in 4-direction, 6-direction, as well as 8-direction sets, as shown in Figure 1. The black “◆” indicates the current location of the robot, and the black “●” indicates the optional target point for the next move. It should be noted that, for the majority of cognitive strategies, the evaluation of the next goal point also includes the current location of the robot; as Entrotaxis is based on the maximum entropy strategy, the current location of the robot is not considered.

**Figure 2:**
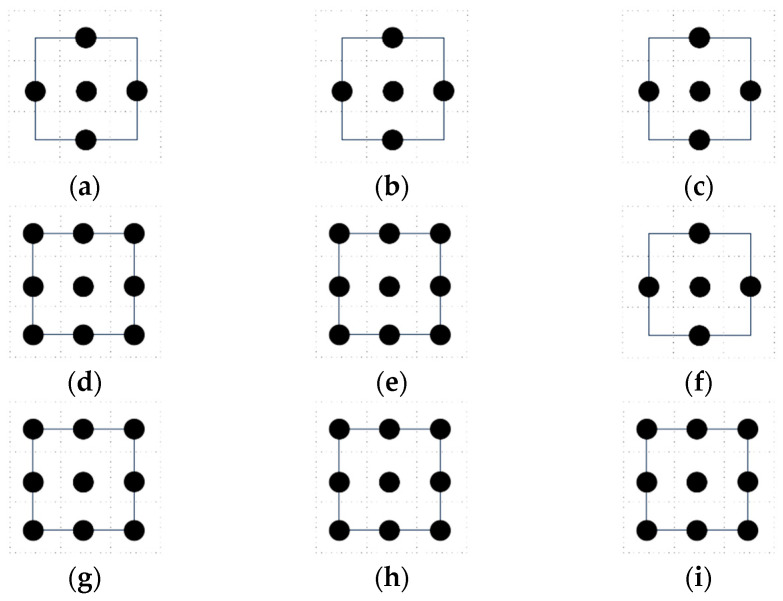
Schematic diagram of admissible action sets in a 3D space: (**a**) 6-direction set, left view; (**b**) 6-direction set, main view; (**c**) 6-direction set, top view; (**d**) 14-direction set, left view; (**e**) 14-direction set, main view; (**f**) 14-direction set, top view; (**g**) 26-direction set, left view; (**h**) 26-direction set, main view; and (**i**) 26-direction set, top view.

In 3D, a set of admissible actions is available in 6-direction, 14-direction, and 26-direction sets. The left, main, and top views of their pathway units are shown in Figure 2. The pathway units can be imagined as within a positive 3D space, and the robot is located at the center of the positive 3D space. Similarly, the black “◆” indicates the current location of the robot, and the black “●” indicates the optional target point for the next move.

## 3. Adaptive Space-Aware Infotaxis II Search Scheme

### 3.1. Construction of Space-Aware Infotaxis II

A very important component of cognitive search strategies is the design of reward functions, which determines how the robot chooses the next moving direction under the current PDF and largely determines the efficiency of OSL. Classical cognitive search strategies introduce information entropy to measure the uncertainty of the environment, which is calculated as shown in Formula (9). Information entropy contains only the mastery of information about the current environment, but does not contain a measure of potential source positions relative to the position of the robot. To improve the exploitation behavior, the distance information metric H was introduced to quantify the distance between the robot and all the potential source positions [39]. The distance information metric is updated according to:

(14) $$H=\sum_{\Omega }{P_{t}(r_{0})||r_{a}-r||}_{1}$$

where $P_{t}(r_{0})$ is the PDF in the current state, $r_{a}$ is all possible source positions, r denotes the current position of the robot, and ${\left||r_{a}-r|\right|}_{1}$ denotes the Manhattan distance between the robot and the potential position (other distance calculations can be used as well, e.g., Euclidean distance and Chebyshev distance). Combining information entropy S and distance information H as a new reward measure to characterize the uncertainty of the environment, denoted as M, the calculation method is as follows:

(15) $$M={log}_{2}(H+2^{S-1}-0.5)$$

Therefore, the space-aware Infotaxis (SAI) [39] reward function is:

(16) $$\Delta M_{1}\left(r\to r_{j}\right)=P_{t}\left(r_{j}\right)\left[-M\right]+\left[1-P_{t}\left(r_{j}\right)\right][p\left(0,r_{j}\right)∆M_{0}+p\left(1,r_{j}\right)∆M_{1}+\cdots +p\left(k,r_{j}\right)∆M_{k}]$$

Suppose the robot arrives at position $r_{j}$ at time t, and its collected information is in $P_{t}(r_{j})$. The first term on the right side of Formula (16) indicates that the robot found the location of the odor source using the current information, which means that $P_{t+1}$ becomes a δ function and the information entropy becomes 0 at the next moment, but this term is meaningful only when the robot finds the source location at the next moment, and the remaining moments have a small value of $P_{t}\left(r_{j}\right)$. The second term on the right side is an exploratory term, which means that the robot did not find the source at $r_{j}$, where $p\left(k,r_{j}\right)$ denotes the probability that the robot makes k detections at position $r_{j}$ in a time interval, given by the Poisson distribution. In this regard, the first term is discarded, and a new reward function is established, which enhances the exploration behavior. The new reward function is:

(17) $$\Delta M_{2}\left(r\to r_{j}\right)=\left[1-P_{t}\left(r_{j}\right)\right][p\left({0,r}_{j}\right)∆M_{0}+p\left(1,r_{j}\right)∆M_{1}+\cdots +p\left({k,r}_{j}\right)∆M_{k}]$$

### 3.2. Adaptive Navigation-Updated Mechanism

To compensate for the performance loss caused by discarding the exploitation term in the reward function, and to better achieve the balance between exploration and exploitation behaviors, this paper proposes an information adaptive navigation-updated mechanism, where the robot relies on the information obtained at each moment to determine the moveable range movement of the next moment. The adaptive change in *movement* is set as follows:

(18) $$movement=s_{0}*s_{f}=s_{0}*\;{\left(S_{max}-S\right)}^{-e}$$

where $s_{f}={\left(S_{max}-S\right)}^{-e}$ denotes the navigation correction factor, S is the information entropy of the current location of the robot, $S_{max}$ is the maximum value of the information entropy of the environment, e denotes the information adaptive parameter, and $s_{0}$ is the initial range of movement according to:

(19) $$s_{0}=speed*dt$$

where speed is the robot’s moving speed and dt is the time for the robot to move one grid. In the case that the initial state is unknown to the whole environment, the unknown situation in each place in the environment is the same; so, the initial distribution is set to a uniform distribution, and the information entropy is maximal. The initial PDF is set to:

(20) $$P_{t\_initial}(r_{0})=\left\{\begin{array}{c} \frac{1}{S_{\Omega }}\;\;\;\;\;\;\;\;\;\;\;\;\;\;\left(x,y\right)\in \Omega \\ 0\;\;\;\;\;\;\;\;\;\;\;\;\;\;\;\mathrm{otherwise} \end{array}\right.$$

where $S_{\Omega }$ is the area of the search region; then, (X, Y) obeys a uniform distribution on Ω, denoted as (X,Y)~U(Ω). Meanwhile, the maximum value of information entropy is found:

(21) $$S_{max}=-\sum_{r_{0}\in \Omega }P_{t\_initial}(r_{0})logP_{t\_initial}(r_{0})$$

**Figure 3:**
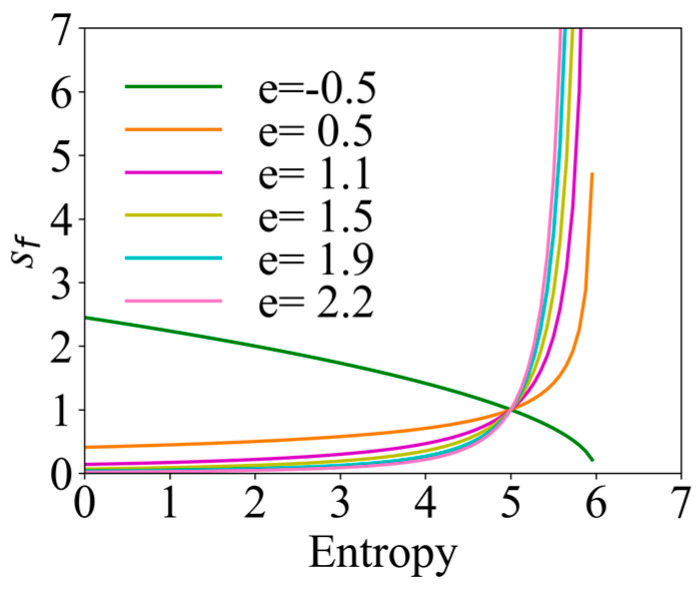
Variation curves of the navigation correction factor under different information adaptive parameters.

In the process of robot searching, the uncertainty of the environment decreases, the overall information entropy shows a decreasing trend, and $\left(S_{max}-S\right)$ gradually increases. The movable range of the robot should be gradually reduced with the searching; so, $s_{f}={\left(S_{max}-S\right)}^{-e}$ was introduced. To better adapt to the changes in the environment, the information adaptive parameter e was introduced, and its variation range was set to [0, 1.5]. Taking the maximum value of information entropy $S_{max}=6.0$ as an example, the changes in the navigation correction factor $s_{f}$ with information entropy under different information adaptive parameter e are shown in Figure 3. Six values of −0.5, 0.5, 1.1, 1.5, 1.9, and 2.2 were selected for e. When the value of e is positive, with the reduction in information entropy, $s_{f}$ gradually decreases, which is in line with the expected design, and the exploration behavior in the early stage is enhanced, and the exploitation behavior in the later stage is enhanced. When the value of e is negative, the trend of the change in $s_{f}$ is just the opposite, which is not in line with the original intention of the design. Therefore, e should be selected as a positive number. However, it should be noted that, if a too large value of e is selected, there will be a situation where the search range is particularly large at the beginning, which may exceed the search range and miss important information about the odor plume; and at a later stage, there can be a situation where the search range is too small, which will result in an excessive exploitation behavior of the robot, which limits the confirmation of the odor source and leads the robot to fall into a local optimum. In this regard, [0, 1.5] was selected as the interval of variation of e.

### 3.3. Finding Optimal Information Adaptive Parameters Based on ACSSA

#### 3.3.1. Multi-Peak Optimization Problem

**Figure 4:**
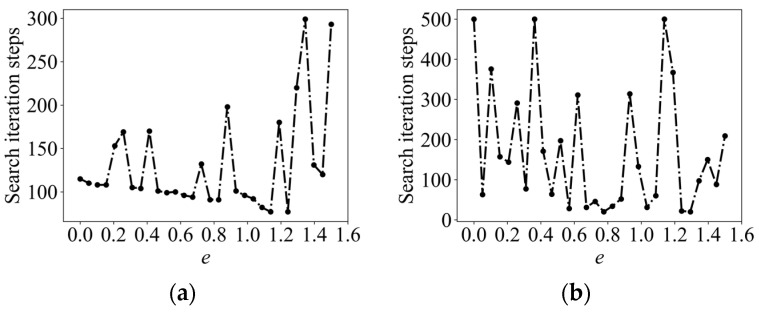
Multi-peak optimization problem: 2D parameters: $D=0.5\;m^{2}/s$, τ=100 s, R=0.6 Hz, V=1 m/s, a=0.1 m, $x\in \left[0,\;9\right]$, y∈[0, 8], and odor source location (1, 6.4); 3D parameters: $D=0.6\;m^{2}/s$, τ=200 s, R=5 Hz, V=1 m/s, a=0.2 m, $x\in \left[0,\;18\right]$, $y\in \left[0,\;18\right]$, $z\in \left[0,\;18\right]$, and odor source location (2, 2, 5). (**a**) The 4-direction set in 2D; and (**b**) 6-direction set in 3D.

The information adaptive parameter e was finely divided in the range of [0, 1.5] by enumeration and the corresponding number of iteration steps was calculated to find out the relatively shortest search path, called enumerating space-aware Infotaxis II (ESAInfotaxis II). Taking the 4-direction set in 2D and the 6-direction set in 3D as an example, the change curve of the number of iterative steps with e is shown in Figure 4. The change in the number of iterative steps does not conform to the law of linear change, whether in 2D or 3D, and the optimal path-solving problem has multiple local optimal values.

To solve the problem of the enumeration method not being able to find the optimal e accurately, a swarm intelligence algorithm is proposed to be used for the solution of the optimal e problem. As it can be seen in Figure 4, the optimal search path-solving problem has multiple local optimal values and only one global optimal value, which is a multi-peak optimization problem. Therefore, a swarm intelligence algorithm that has a strong global search ability and does not easily fall into the local optimum was considered.

**Figure A1:**
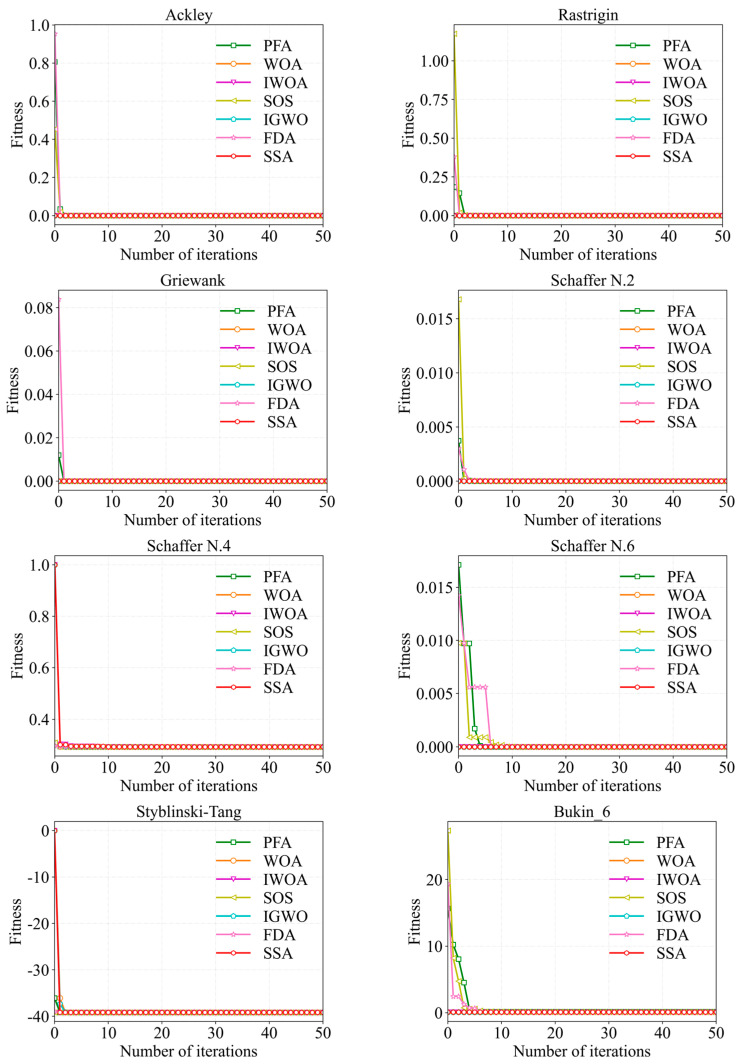
Convergence curves of the swarm intelligence algorithms.

The comparison process of 20 swarm intelligence algorithms under multi-peak test functions can be found in Appendix A. The specific information of multi-peak test functions is shown in Table A1, and the parameters of 20 swarm intelligence algorithms are shown in Table A2. It can be seen in Table A3 and Figure A1 that the salp swarm algorithm (SSA) shows significant competitiveness and stability on the multi-peak test functions with respect to both mean, std as well as time. SSA always maintains a fast convergence speed as well as a high convergence accuracy compared to other algorithms. In this regard, SSA was finally used in this paper for solving the optimal e problem.

#### 3.3.2. Adaptive Cosine Salp Swarm Algorithm

**Figure 5:**
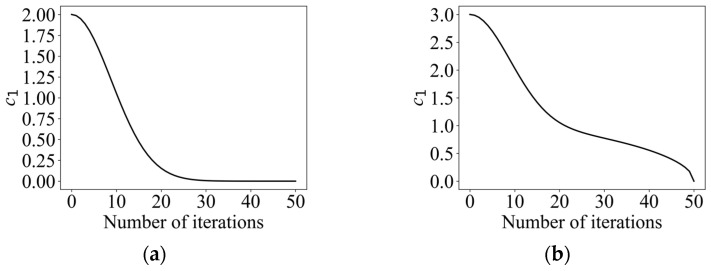
(**a**) Convergence factor curve for a conventional scheme. (**b**) Cosine convergence factor variation curve.

SSA simulates the foraging and navigational behaviors of salps in the ocean. Salps behave as a chain when searching for food; the closest salp to the food serves as the leader, called the head of the chain, and the rest of the salps serve as the followers. Each of the followers approaches toward the previous salp, and this chain of salps searches for the food source F in the search space. Since the number of variables in this paper is only one parameter of the information adaptive parameter, the leader position of the SSA is updated as follows:

(22) $$x^{1}=\left\{\begin{array}{c} F+c_{1}\left(\left(ub-lb\right)c_{2}+lb\right),c_{3}\geq 0.5\\ F-c_{1}\left(\left(ub-lb\right)c_{2}+lb\right),c_{3}<0.5 \end{array}\right.$$

where $x^{1}$ denotes the location of the leader; F denotes the location of the food source, and the optimal solution is assigned to F based on the a fitness function; ub and lb are the upper and lower bounds of the search space, respectively; $c_{1}$ is the main parameter for balancing the global exploration and the local exploitation, which is determined by Equation (23); and $c_{2}\;and\;c_{3}$ are random numbers generated in the interval [0, 1].

(23) $$c_{1}=2e^{-{\left(4d/d_{max}\right)}^{2}}$$

where d and $d_{max}$ denote the current iteration number and the maximum iteration number, respectively. The $c_{1}$ change curve is shown in Figure 5a. Since solving the optimal path is a multi-peak problem, the algorithm is required to have a strong ability to jump out of the local optimum. Therefore, the cosine control factor was introduced in this paper, which makes the range of the convergence factor become larger, while the decreasing trend becomes slower, and maintains a relatively high global exploration ability at the early stage. $c_{1}$, the improved convergence factor, is calculated as shown in Equation (24), and its change curve is shown in Figure 5b.

(24) $$c_{1}=2e^{-{\left(4d/d_{max}\right)}^{2}}+\sqrt{cos((\pi d)/(2d_{max}))}$$

The follower position update utilizes Newton’s laws of motion as follows:

(25) $$x^{i}=\frac{x^{i}+x^{i-1}}{2},i\geq 2$$

where i≥2 and $x^{i}$ denotes the position of the *i*th salp follower. Ultimately, the chain of salps can be modeled by Formula (22) and Formula (25).

**Figure 6:**
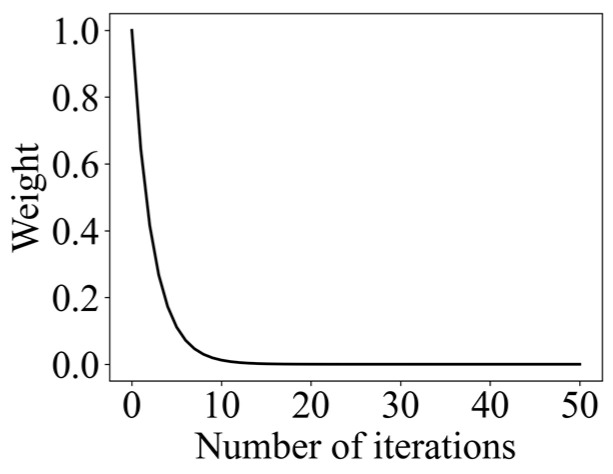
Adaptive weight change curve.

Moreover, an adaptive weight ω was utilized to control the leader’s search range, as shown in Formula (26), and its change curve is shown in Figure 6. As it can be seen in Figure 6, making the leader search range larger at the beginning helps to enhance the exploration behavior, which improves the convergence speed of the algorithm. As the search proceeds, the leader search range gradually becomes smaller. In the late iteration, the weight is lower, which makes the leader carry out a locally accurate search in the vicinity of the optimal solution, which improves the ability to find the optimal solution. After adding adaptive weights, the position of the leader is updated as expressed in Formula (27).

(26) $$\omega =3^{\left(-20d/d_{max}\right)}$$

(27) $$x^{1}=\left\{\begin{array}{c} F+{\omega c}_{1}\left(\left(ub-lb\right)c_{2}+lb\right),c_{3}\geq 0.5\\ F-\omega c_{1}\left(\left(ub-lb\right)c_{2}+lb\right),c_{3}<0.5 \end{array}\right.$$

Ultimately, we combined the improved reward function SAInfotaxis II and adaptive navigation-updated mechanism with the improved ACSSA to adaptively find the optimal information adaptive parameter e, so as to find the shortest search path in a specific scenario, which is called adaptive space-aware Infotaxis II (ASAInfotaxis II).

## 4. Simulations and Discussion

The section analyzes and compares the performance of ASAInfotaxis II with the classical Infotaxis, Infotaxis II, Entrotaxis, Sinfotaxis, and space-aware Infotaxis for OSL using sparse cues through numerical simulation experiments in 2D as well as 3D to verify the feasibility and effectiveness of ASAInfotaxis II. The experimental simulation environment was Windows 11 64-bit, PyCharm2020, Intel Core i7-12700H processor, and 16 GB RAM. In this paper, the algorithms were evaluated according to the following aspects:

(1) The number of search iteration steps: The sum of steps moved by the robot to complete the search task. One of the moving steps refers to the whole process of the robot staying at the original position, updating the PDF, making a moving decision, and moving to the next target point. The number of iterative steps is the basic index to measure the efficiency of the search methods.
(2) The time to find the source: Since ESAInfotaxis II and ASAInfotaxis II are not fixed-step searches, the evaluation metric of the search time was added to further judge the search efficiency of the algorithms.
(3) Information collection rate: The change in information entropy with the number of search steps in the source search process. The change in information entropy reflects the collection of environmental information in the robot search process in real time.
(4) PDFs of the arrival times: The variation in PDFs with the search time; arrival time pdfs can respond to the ability to find the source of robots.

The robot terminates the search when any of the following conditions are met:

(1) Search iteration steps of the robot reach 500, but the odor source is not found.
(2) The robot is considered to have found the odor source if its distance from the source is within the specified range src_radius.

### 4.1. Simulations for Two-Dimensional Scenarios

#### 4.1.1. Two-Dimensional Simulation Scenario

Since the odor information in a natural scene is sparse and discontinuous, this paper tried to fit the actual application scene as much as possible to achieve OSL in a sparse environment. As it can be seen in the Beaufort scale, the wind speed below 0.2 m/s can be regarded as “no wind (calm)”; so, this paper set the wind speed to 1 m/s to achieve the conditions of sparse odor cues. The search radius of the robot was a = 0.1 m, which is in line with the general size of ground robots in real scenarios. The simulated spatial extent was set to 9 m × 8 m, and the gird size was set to 0.1 m × 0.1 m due to the small size of the 2D scene. The effective diffusion coefficient of the plume was $D=0.5\;m^{2}/s$, the particle lifetime was τ=100 s, and the odor source emitted at a rate of R=0.6 Hz at the location of (8, 7.4). The robot moved at a speed of 0.1 m/s, and its sensors detected at every 1 s. The initial position of the robot was arbitrarily specified. The sensor detection threshold was th=0.005, above which the sensing result was “1”. When the position of the robot and the odor source was within the range of $src_{radius}=0.2\;m$, the robot was considered to have found the odor source.

#### 4.1.2. Two-Dimensional Simulation Results

The results of the mean iterative steps after 50 simulations of randomly selecting the initial position of the robot with different movement strategies in 2D scenarios, as set in Section 4.1.1, are shown in Table 1. As it can be seen in Table 1, ASAInfotaxis II, proposed in this paper, shows a very significant improvement in search efficiency compared to the classical cognitive search strategies. The improvement in the search efficiency of SAInfotaxis II using the improved reward function alone is unstable, and the mean iterative steps in the 6-direction set exceed those of the classical strategies, which may be due to the strong exploration behavior that makes the robot fall easily into a local optimal state and “passes by” the odor source. The adaptive navigation-updated mechanism compensates for this by changing the movement range of the robot in real time through information entropy, which becomes relatively small in the later stages of the search, thus making the robot less likely to fall into a local optimum in the source confirmation stage and achieving a balance between the exploration and exploitation behaviors. The search efficiency of ESAInfotaxis II using the enumeration method is also significantly improved, but the comparison with ASAInfotaxis II shows that the optimal e cannot be found precisely using the enumeration method, and the search efficiency is further improved by solving e with ACSSA.

**Figure 7:**
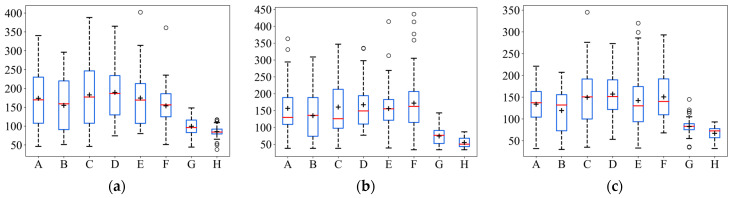
Box line plots of ASAInfotaxis II with the other cognitive search strategies for various admissible action sets in 2D scenarios, where A = Infotaxis, B = Infotaxis II, C = Entrotaxis, D = Sinfotaxis, E = Space-aware Infotaxis, F = SAInfotaxis II, G = ESAInfotaxis II, and H = ASAInfotaxis II. (**a**) The 4-direction; (**b**) 6-direction; and (**c**) 8-direction sets. The black “+” indicates the mean of the data, the red line indicates the median of the data, and the black “○” indicates outliers.

In order to more intuitively represent the numerical distribution of all algorithms, box line plots were used to represent the distribution of each algorithm result in terms of the iterative steps searched under the different initial positions of the robot. The distribution of the search results for the eight strategies is shown in Figure 7. In Figure 7, it can be seen that ASAInfotaxis II shows remarkable stability and excellent search performance under all admissible action sets, and its mean, upper edge, upper quartile, lower edge, lower quartile, and median are consistently lower than those of the other cognitive strategies under the same admissible action set.

**Figure 8:**
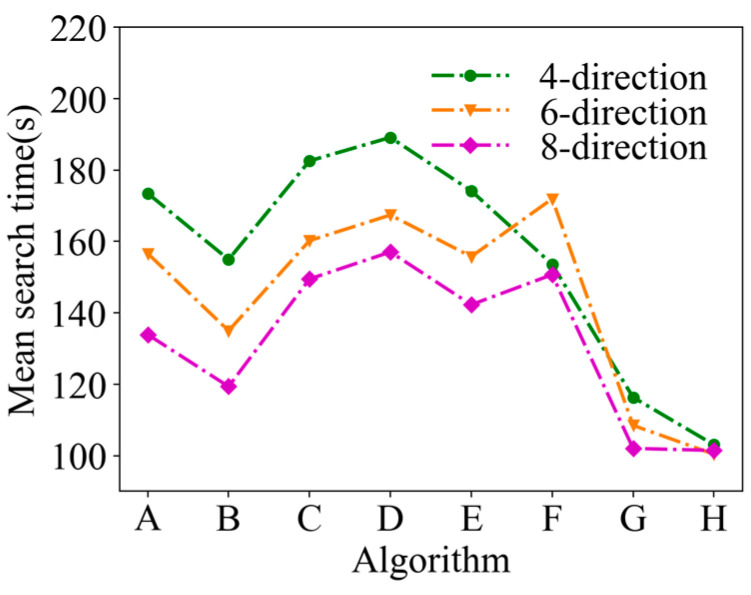
Mean search time in a 2D scene, where A = Infotaxis, B = Infotaxis II, C = Entrotaxis, D = Sinfotaxis, E = Space-aware Infotaxis, F = SAInfotaxis II, G = ESAInfotaxis II, and H = ASAInfotaxis II.

A comparison of the mean search time after 50 simulations under the same conditions is shown in Figure 8. In Figure 8, it can be seen that the mean search time of ASAInfotaxis II remains the lowest under any admissible action set, which indicates a significant improvement in the search efficiency of ASAInfotaxis II.

**Figure 9:**
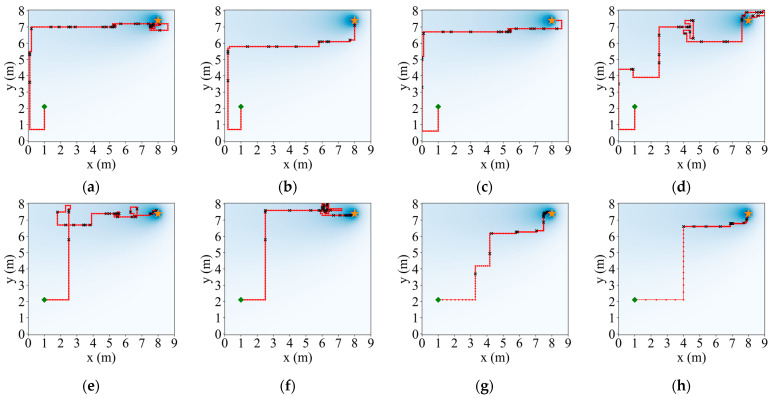
Comparison results of the search paths under a 4-direction admissible action set. (**a**) Infotaxis: 209 steps; (**b**) Infotaxis II: 167 steps; (**c**) Entrotaxis: 187 steps; (**d**) Sinfotaxis: 267 steps; (**e**) Space-aware Infotaxis: 195 steps; (**f**) SAInfotaxis II: 199 steps; (**g**) ESAInfotaxis II: 118 steps; and (**h**) ASAInfotaxis II_ 96 steps. The orange star indicates the true source location, the green square indicates the initial robot position, the red line indicates the trajectory of the robot, red dots indicate zero measurements, and black crosses indicate non-zero measurements.

Taking the 4-direction admissible action set as an example, the initial position (1, 2.1) of the robot was randomly selected to make a comparison of the search paths, as shown in Figure 9. In Figure 9, it can be seen that Infotaxis, Sinfotaxis, and space-aware Infotaxis fall into a local optimum during the process. When the new improved SAInfotaxis II reward function is used alone, the robot may fall into a local optimum at a later stage of the search due to an enhanced exploratory behavior, as shown in Figure 9f, but when SAInfotaxis II is combined with the adaptive navigation-updated mechanism, the narrowing of the movement range at a later stage neutralizes well the over-exploration behavior caused by the reward function, and the final search efficiency and search behavior are considerably improved after the optimization by ACSSA, as shown in Figure 9h.

**Figure 10:**
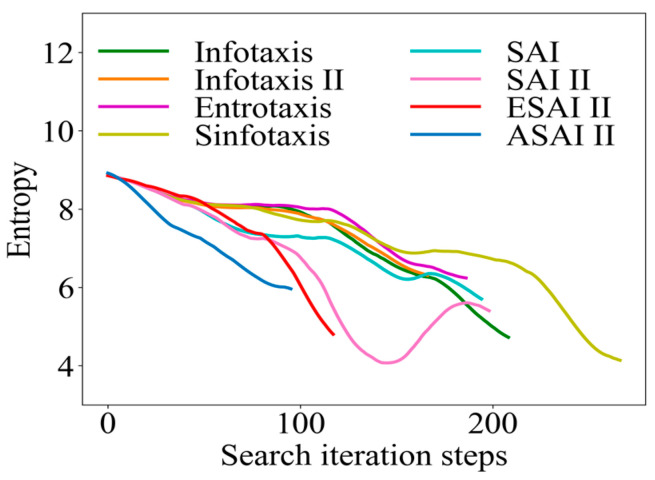
Comparison of the information-gathering rate curves in a 2D scene, where SAInfotaxis II = SAI II, ESAInfotaxis II = ESAI II, and ASAInfotaxis II = ASAI II.

Similarly, taking the 4-direction admissible action set as an example, the initial position of the robot (1, 2.1) was selected to obtain the information collection rate curve, as shown in Figure 10. As mentioned in Section 3.1, when the robot locates the odor source, the information entropy becomes 0. Therefore, we do not show the moment when the final information entropy becomes 0, but focused on analyzing the change in information entropy in the search process. The degree of inclination of the curve reflects the information collection rate of the algorithms. As it can be seen in Figure 10, with the improved SAInfotaxis II reward function alone, the exploratory behavior of the robot is significantly improved compared to several other classical strategies, but the information entropy of such a scheme appears to have rebounded in the later stages of the search, which indicates that the robot is trapped in a local optimum. After the introduction of the adaptive navigation-updated mechanism, this shortcoming is compensated well. Finally, after the optimization by ACSSA, the exploration behavior of ASAInfotaxis II always maintains a significant advantage, and the exploration and exploitation behaviors always maintain a relatively good balance in the whole search process.

**Figure 11:**
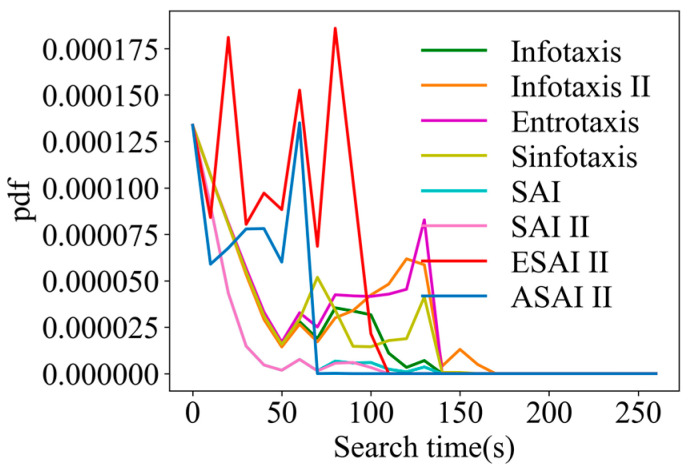
Comparison of the arrival time pdfs in a 2D scene, from the point (7, 4) for ASAInfotaxis II and several cognitive strategies, where SAInfotaxis II = SAI II, ESAInfotaxis II = ESAI II, and ASAInfotaxis II = ASAI II.

In a 2D scene, a randomly selected fixed point (7, 4) is used to make a curve comparison of the arrival time pdf, as shown in Figure 11. In Figure 11, it can be seen that the several types of classical cognitive search strategies have a poor convergence ability with very slowly decaying tails, especially Infotaxis II, which is due to the fact that only the exploration term is preserved, leading to too much greed. Although only the exploration term is preserved in the novel SAInfotaxis II reward function, distance information is introduced, which effectively enhances the exploitation behavior, and thus this strategy decays much faster. After combining the adaptive navigation-updated mechanism as well as ACSSA on top of SAInfotaxis II, ASAInfotaxis II has a much shorter arrival time with only a relatively short time to converge, indicating that the robot is relatively better at source finding.

### 4.2. Simulations for Three-Dimensional Scenarios

#### 4.2.1. Three-Dimensional Simulation Scenario

Considering a realistic scene as well as a sparse environment setting, the wind speed was 1 m/s, and the robot search radius was a = 0.2 m, which is in line with the general size of the UAV under actual circumstances. The simulated spatial range was set to 18 m × 18 m × 18 m, and the gird size was set to 1 m × 1 m × 1 m because the search range in 3D is larger than that of the scene in 2D. The effective diffusion coefficient of the plume was $D=0.6\;m^{2}/s$, the particle lifetime was τ=200 s, and the odor source emitted at a rate of R=5 Hz at the location of (2, 2, 5). The robot moved at a speed of 1 m/s, and its sensors detected at every 1 s. The initial position of the robot was arbitrarily specified. The sensor detection threshold was th=0.005, above which the sensing result was “1”. When the position of the robot and the odor source were within the range of src_radius=1 m, the robot was considered to have found the odor source.

#### 4.2.2. Three-Dimensional Simulation Results

The results of the mean iterative steps after 50 simulations of randomly selecting the initial position of the robot with different admissible action sets in 3D, as set in Section 4.2.1, are shown in Table 2. In Table 2, ASAInfotaxis II, proposed in this paper, shows a very significant improvement in search efficiency compared to the classical strategies. Similarly, as in the 2D scene, the improvement in search efficiency using SAInfotaxis II alone is erratic, and its mean iterative steps exceed that of the classical strategies under various admissible action sets, which is well compensated for by the adaptive navigational-updating mechanism that achieves a balance between exploration and exploitation behaviors. The search efficiency of ESAInfotaxis II using the enumeration approach is also improved significantly, but the comparison with ASAInfotaxis II shows that the optimal e cannot be found accurately by using the enumeration approach, and ACSSA for solving e leads to a further improvement in the search efficiency.

**Figure 12:**
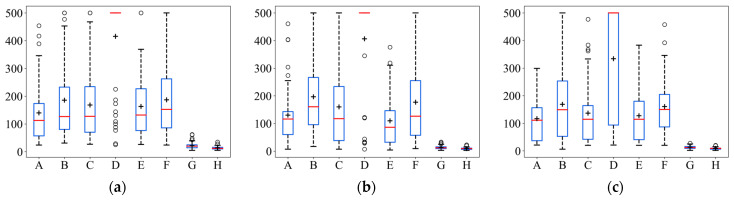
Box line plots of ASAInfotaxis II with the other cognitive search strategies for various admissible action sets in 3D scenarios, where A = Infotaxis, B = Infotaxis II, C = Entrotaxis, D = Sinfotaxis, E = Space-aware Infotaxis, F = SAInfotaxis II, G = ESAInfotaxis II, and H = ASAInfotaxis II. (**a**) The 6-direction set; (**b**) 14-direction set; and (**c**) 26-direction set. The black “+” indicates the mean of the data, the red line indicates the median of the data, and the black “○” indicates outliers.

Similarly, in order to visually represent the numerical distribution of the search iterative steps for all algorithms in 3D scenarios, box line plots were made, as shown in Figure 12. In Figure 12, it can be seen that ASAInfotaxis II shows the same remarkable stability and excellent search performance under all the admissible action sets, more so than in 2D scenarios. Its mean, upper edge, upper quartile, lower edge, lower quartile, and median consistently outperform those of the other cognitive search strategies.

**Figure 13:**
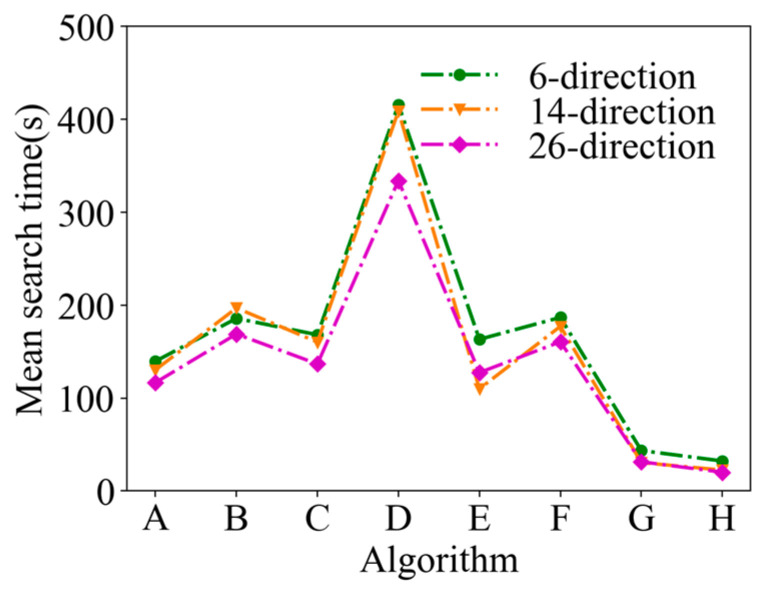
Mean search time in 3D scenarios, where A = Infotaxis, B = Infotaxis II, C = Entrotaxis, D = Sinfotaxis, E = Space-aware Infotaxis, F = SAInfotaxis II, G = ESAInfotaxis II, and H = ASAInfotaxis II.

A comparison of the mean search time after 50 simulations under the same conditions is shown in Figure 13. In Figure 13, it can be seen that the mean search time of ASAInfotaxis II remains the lowest under any admissible action set, and the improvement in search efficiency is more pronounced than that in 2D scenarios.

**Figure 14:**
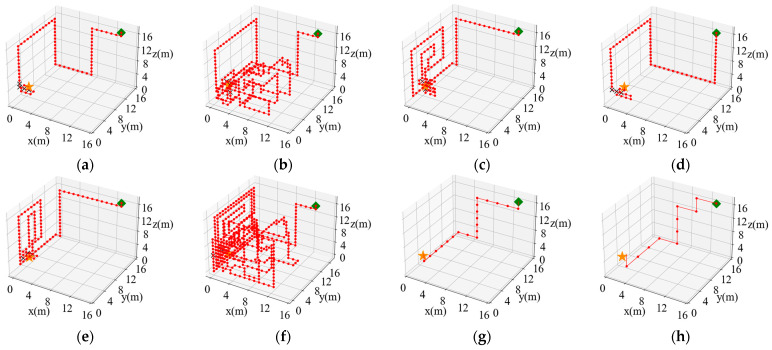
Comparison results of the search paths under the 6-direction admissible action set. (**a**) Infotaxis: 77 steps; (**b**) Infotaxis II: 283 steps; (**c**) Entrotaxis: 129 steps; (**d**) Sinfotaxis: 81 steps; (**e**) Space-aware Infotaxis: 113 steps; (**f**) SAInfotaxis II: 500 steps; (**g**) ESAInfotaxis II: 21 steps; and (**h**) ASAInfotaxis II: 12 steps. The orange star indicates the true source location, the green square indicates the initial robot position, the red line indicates the trajectory of the robot, red dots indicate zero measurements, and black crosses indicate non-zero measurements.

Taking the 6-direction admissible action set as an example, the initial position of the robot (14, 14, 17) was randomly selected to make a comparison of the search paths, as shown in Figure 14. In Figure 14, it can be seen that the classical cognitive search strategies all fall into a local optimum during the process. When using the newly improved SAInfotaxis II reward function alone, the robot also falls into a local optimum due to an enhanced exploration behavior, as shown in Figure 14f, but when SAInfotaxis II is combined with the adaptive navigation-updated mechanism and through the ACSSA optimization search, the search efficiency as well as behavior are considerably improved, as shown in Figure 14h.

**Figure 15:**
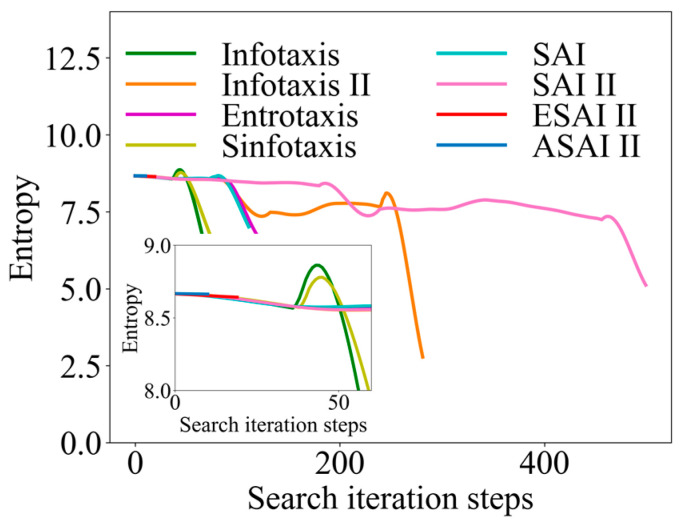
Comparison of information-gathering rate curves in 3D scenarios, where SAInfotaxis II = SAI II, ESAInfotaxis II = ESAI II, and ASAInfotaxis II = ASAI II.

Similarly, taking the 6-direction admissible action set as an example, the initial position of the robot (14, 14, 17) was randomly selected to obtain the information collection rate curve, as shown in Figure 15. Similarly, the moment when the final information entropy becomes 0 was not represented in this paper, focusing on analyzing the change in information entropy during the search process. In Figure 15, it can be seen that, in the classical strategies, the information entropy almost always appears rebounded, which indicates that the robot may fall into a local optimum in the search. However, ASAInfotaxis II avoids this well, and ASAInfotaxis II achieves OSL with only a very small entropy drop, which indicates that this scheme can achieve OSL in the case of not having much information about the environment.

**Figure 16:**
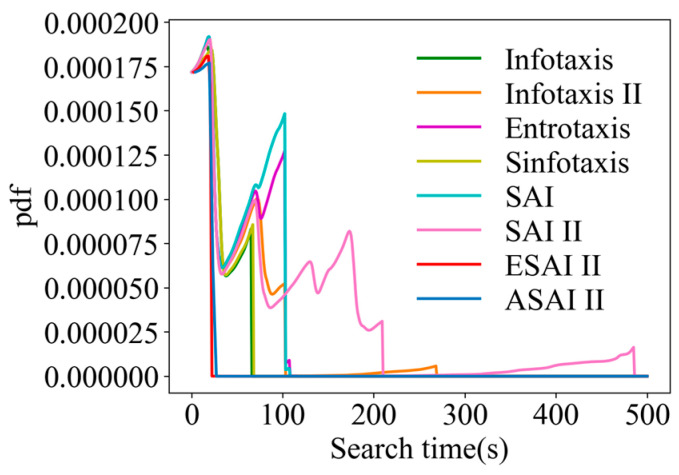
Comparison of the arrival time pdfs in 3D scenarios, from the point (6, 14, 5) for ASAInfotaxis II and several cognitive strategies, where SAInfotaxis II = SAI II, ESAInfotaxis II = ESAI II, and ASAInfotaxis II = ASAI II.

A comparison of the arrival time pdfs is shown in Figure 16 for a randomly selected fixed point (6, 14, 5) in 3D scenarios. From Figure 16, it can be seen that SAInfotaxis II as well as Infotaxis II converge very slowly due to over-exploration. In contrast, the performance of ASAInfotaxis II is improved after the inclusion of the adaptive navigation-updated mechanism, which balances the exploration and exploitation behaviors of the robot relatively well.

## 5. Conclusions

Aiming at the problems of the low search efficiency of the current OSL strategies and the imbalance between exploration and exploitation behaviors that cause the robot to easily fall into local optima, ASAInfotaxis II is proposed. ASAInfotaxis II introduces three strategies based on Infotaxis. First, a new reward function is designed, which takes into account distance information and emphasizes the exploration behavior of the robot. Second, the movement range of the robot is adjusted in real time through an adaptive navigation-updated mechanism to avoid the robot falling into a local optimum. Third, ACSSA is used to confirm the optimal information adaptive parameters. Comparative simulation experiments between ASAInfotaxis II and the classical search strategies were conducted in 2D and 3D scenarios with respect to search efficiency and search behavior. The experimental results show that ASAInfotaxis II can quickly and accurately locate the odor source, and the number of iterative search steps in 2D scenarios is reduced by more than 44.96% compared to the classical cognitive strategies, and the number of iterative search steps in 3D scenarios is reduced by more than 90.17% compared to the classical cognitive strategies. The mean search time is also significantly reduced. ASAInfotaxis II demonstrates a good searching ability as well as stability, especially in 3D scenarios, which shows excellent performance and obvious competitiveness. In addition, the information collection rate of ASAInfotaxis II is maintained at a faster rate, implying that the robot has a significant advantage in the collection of environmental information as well as the process of converging to the source of the odor at a later stage. The implementation results show that ASAInfotaxis II can accomplish the efficient localization of odor sources in complex environments, such as rich in local information and with sparse cues.

In future work, we will optimize the search model to make fuller use of the prior information for further narrowing down the possible source locations, thus saving computational costs and speeding up the convergence of the algorithm. It is also planned to extend the OSL problem to more complex scenarios, such as near streets or around buildings.

## Figures and Tables

**Table 1:** Comparison of the mean search iteration steps of different algorithms in 2D scenarios.

Algorithms	4-Direction Set	6-Direction Set	8-Direction Set
Infotaxis	173.48	156.64	133.92
Infotaxis II	154.94	134.98	119.36
Entrotaxis	182.56	160.22	149.48
Sinfotaxis	189.14	167.40	157.04
Space-aware Infotaxis	174.16	155.76	142.28
SAInfotaxis II	153.58	171.94	150.70
ESAInfotaxis II	99.28	74.66	82.52
ASAInfotaxis II	85.28	55.18	66.92

**Table 2:** Comparison of the mean search iteration steps of the different algorithms in 3D scenarios.

Algorithms	6-Direction Set	14-Direction Set	26-Direction Set
Infotaxis	139.60	130.34	116.66
Infotaxis II	185.72	196.88	168.88
Entrotaxis	168.06	160.22	136.66
Sinfotaxis	415.76	408.83	334.04
Space-aware Infotaxis	163.18	109.90	127.40
SAInfotaxis II	186.74	177.12	160.54
ESAInfotaxis II	21.56	13.44	13.32
ASAInfotaxis II	13.72	9.14	8.54

## Data Availability

The dataset generated from the numerical simulation experiments in this paper can be shared by emailing 202121902015@stu.hebut.edu.cn.

## Appendix A

In this appendix, we provide specific information about 8 multi-peak test functions selected from CEC (International Conference on Evolutionary Computation), as shown in Table A1.

**Table A1:** Description of the 8 classic benchmarking functions.

	Function	Dim	Range	Optimum Value
Ackley	$f\left(x\right)=-20*\exp\left(-0.2\sqrt{\frac{1}{n}\sum_{i}^{n}x_{i}^{2}}\right)-\exp\left(\frac{1}{n}\sum_{i=1}^{n}\cos\left(2\pi x_{i}\right)\right)+20+e$	1	[−32, 32]	0
Rastrigin	$f\left(x\right)=10n+\sum_{i=1}^{n}\left[x_{i}^{2}-10cos(2\pi x_{i})\right]$	1	[−5.12, 5.12]	0
Griewank	$f\left(x\right)=1+\frac{1}{4000}\sum_{i=1}^{n}x_{i}^{2}-\prod_{i=1}^{n}cos(\frac{x_{i}}{\sqrt{i}})$	1	[−600, 600]	0
Schaffer N.2	$f\left(x\right)=0.5+\frac{{sin}^{2}\left(x_{1}^{2}-x_{2}^{2}\right)-0.5}{{\left[1+0.001(x_{1}^{2}+x_{2}^{2})\right]}^{2}}$	2	[−100, 100]	0
Schaffer N.4	$f\left(x\right)=0.5+\frac{{cos}^{2}\left({sin(|x}_{1}^{2}-x_{2}^{2}|\right))-0.5}{{\left[1+0.001(x_{1}^{2}+x_{2}^{2})\right]}^{2}}$	2	[−100, 100]	0.292579
Schaffer N.6	$f\left(x\right)=0.5+\frac{{sin}^{2}\left(\sqrt{x_{1}^{2}+x_{2}^{2}}\right))-0.5}{{\left[1+0.001(x_{1}^{2}+x_{2}^{2})\right]}^{2}}$	2	[−100, 100]	0
Styblinski–Tang	$f\left(x\right)=0.5*\sum_{i=1}^{n}\left(x_{i}^{4}-16x_{i}^{2}+5x_{i}\right)$	1	[−5, 5]	−39.16599
Bukin_6	$f\left(x\right)=100\sqrt{\left|x_{2}-0.01x_{1}^{2}\right|}+0.01|x_{1}+10|$	2	$x_{1}\epsilon [-15,-5]$ $x_{2}\epsilon [-3,3]$	0

A comparison of 20 swarm intelligence algorithms was carried out, and the parameters in each algorithm were set for the original document, and the specific parameter settings are shown in Table A2.

The test results for 20 swarm intelligence algorithms running independently for 30 times on 8 benchmark test functions are shown in Table A3. It should be noted that, in this paper, the mean results of Schaffer N.4 are accurate to 0.000001; the mean results of Styblinski–Tang are accurate to 0.00001; and the test results are accurate to 0.0001 in the rest of the cases. In Table A3, PFA, WOA, IWOA, SOS, SSA, FDA, and IGWO show significant competitiveness under all the tested functions, and the performances of both mean and std have strong advantages in finding the global optimum as well as jumping out of the local optimum point, contrasting with the other algorithms. The WOA, IWOA, SOS, and IGWO algorithms were solved to the theoretical optimum in the Ackley, Rastrigin, Griewank, Schaffer N.2, and Schaffer N.6 function tests.

In terms of computational costs, MBO has the best performance. In addition, the PSO, SSA, MVO, MBO, and AOA algorithms also have a relatively short running time, while the PFA, FDA, and DA algorithms require large computations with relatively a high time cost, especially the PFA and FDA algorithms, whose computational cost reaches about one minute, although the PFA and FDA algorithms perform better in terms of global searching and circumventing local optimums, but this comes at the cost of a large amount of time.

**Table A2:** Parameter settings of each algorithm.

Algorithm	Specific Parameter Settings
PSO	$c_{1}=2\;c_{2}=2$
PFA	$a=u_{1}*exp(\frac{-2t}{T_{max}})$ $u_{1}\epsilon [-1,1]$
WOA	$a=2-\frac{2t}{T_{max}}$
IWOA	$a=2-\frac{2t}{T_{max}}$
HHO	$a=2-\frac{2t}{T_{max}}$
SOS	${BF}_{1}=1\;or\;2$ ${BF}_{2}=1\;or\;2$
SCA	$r=2-\frac{2t}{T_{max}}$
SSA	$c_{1}=2*exp(-{\left(4*(\frac{t}{T_{max}})\right)}^{2})$
MVO	$a=0.2+\frac{0.8t}{T_{max}}$
SPBO	k=1 or 2
MBO	p=5/12 peri=1.2 BAR=5/12 $S_{max}=1$
JSO	$a=\left(1-\frac{t}{T_{max}}\right)*(2*rand-1)$
IGWO	$a=2-\frac{2t}{T_{max}}$
FDA	β=8
DA	β=1.5 $\delta ={\left(\frac{\Gamma \left(1+\beta \right)*sin(\frac{\pi \beta }{2})}{\Gamma \left(\frac{1+\beta }{2}\right)*\beta *2^{\frac{\beta -1}{2}}}\right)}^{\frac{1}{\beta }}$
ALO	$I=10^{\omega }\frac{t}{T_{max}}$
AOA	α=0.5 $MOA=0.2+\frac{0.8t}{T_{max}}$ $MOP=1-\frac{t^{1/\alpha }}{\left(T_{max}^{1/\alpha }\right)}$
CS	α=0.01 λ=1.5
GOA	$c=c_{max}-t*(\frac{c_{max}-c_{min}}{T_{max}})$ $c_{max}=1$ $c_{min}=0.00004$
GA	mutation_rate=0.1

It is clear from the above analysis that the PFA, WOA, IWOA, SOS, SSA, IGWO, as well as FDA algorithms show significant advantages in solving the mean and std; so, we next analyzed the convergence speed as well as the convergence ability of the above algorithms only. The convergence curves of the above algorithms on the 8 benchmark functions are shown in Figure A1. In Figure A1, it can be seen that the convergence speed and the convergence accuracy of SOS, PFA, and FDA generally perform poorly. Moreover, although WOA and IWOA also have significant advantages in convergence speed, their convergence accuracy is lower than that of SSA on the Schaffer N.4 and Styblinski–Tang.

**Table A3:** Test results of the swarm intelligence algorithms on the benchmark test functions..

Function	Criteria	PSO	PFA	WOA	IWOA	HHO	SOS	SCA	SSA	MVO	SPBO
Ackley	Mean	0.0089	0.0	0.0	0.0	0.0	0.0	0.0	0.0	0.0006	0.0575
	Std	0.0139	0.0	0.0	0.0	0.0001	0.0	0.0	0.0	0.0007	0.2132
	Time	2.5075	87.3500	2.7731	6.2540	3.0480	5.2511	2.8093	2.5220	1.9123	4.3905
Rastrigin	Mean	0.0013	0.0	0.0	0.0	0.0	0.0	0.0	0.0	0.0	0.2839
	Std	0.0029	0.0	0.0	0.0	0.0	0.0	0.0	0.0	0.0	0.6607
	Time	2.3012	78.0795	2.3197	5.7256	2.7999	3.6491	2.4768	2.3112	1.6072	3.8947
Griewank	Mean	0.0	0.0	0.0	0.0	0.0	0.0	0.0	0.0	0.0	0.0001
	Std	0.0	0.0	0.0	0.0	0.0	0.0	0.0	0.0	0.0	0.0005
	Time	2.1831	77.9928	2.4811	5.7041	2.8670	4.4761	2.3947	2.3123	1.2743	3.9660
Schaffer N.2	Mean	0.0	0.0	0.0	0.0	0.0010	0.0	0.0	0.0	0.0002	0.0067
	Std	0.0001	0.0	0.0	0.0	0.0016	0.0	0.0	0.0	0.0012	0.0082
	Time	2.2509	80.7760	2.6066	5.8712	2.7963	4.7656	2.5900	2.1123	1.7282	4.1343
Schaffer N.4	Mean	0.292761	0.292591	0.292658	0.292587	0.293011	0.292580	0.292655	0.292619	0.292586	0.294534
	Std	0.0002	0.0	0.0001	0.0	0.0007	0.0	0.0001	0.0001	0.0	0.0020
	Time	2.4200	81.782	2.5146	5.8164	2.8567	4.8856	2.6282	2.2947	1.7542	4.1134
Schaffer N.6	Mean	0.0084	0.0056	0.0	0.0	0.0071	0.0	0.0053	0.0	0.0094	0.0113
	Std	0.0029	0.0047	0.0	0.0	0.0043	0.0	0.0047	0.0	0.0017	0.0059
	Time	2.2076	82.7493	2.4682	5.5752	2.6915	4.3382	2.5646	2.3579	1.7895	4.1644
Styblinski–Tang	Mean	−39.16609	−39.16617	−39.16617	−39.16617	−39.16616	−39.16617	−39.16601	−39.16617	−38.69494	−39.13254
	Std	0.0001	0.0	0.0	0.0	0.0	0.0	0.0002	0.0	2.5376	0.1102
	Time	2.0835	72.2040	2.0767	5.3305	2.8530	4.1391	2.3961	2.1449	1.5155	3.9183
Bukin_6	Mean	4.5934	0.0955	0.1	0.1	1.3550	0.1038	0.6841	0.1	1.0947	5.7670
	Std	2.7638	0.0330	0.0	0.0	1.3874	0.0183	0.7055	0.0	0.4910	7.2578
	Time	0.8791	23.3484	0.9719	2.3828	1.1133	1.8759	1.0460	0.9330	0.7453	1.6423
**Function**	**Criteria**	**MBO**	**JSO**	**IGWO**	**FDA**	**DA**	**ALO**	**AOA**	**CS**	**GOA**	**GA**
Ackley	Mean	0.0040	0.0032	0.0	0.0	0.0011	0.0	1.5654	0.3197	0.0002	0.0001
	Std	0.0055	0.0029	0.0	0.0	0.0024	0.0	1.4184	0.4081	0.0008	0.0001
	Time	1.6513	2.6549	7.2145	63.1317	38.0546	20.7061	2.6846	4.9786	13.4924	4.2537
Rastrigin	Mean	0.1693	0.0007	0.0	0.0	0.0	0.0	1.1856	0.4213	0.0995	0.0466
	Std	0.3699	0.0009	0.0	0.0	0.0001	0.0	1.2042	0.4773	0.2985	0.1838
	Time	1.3953	2.1311	6.4451	59.1630	36.9516	14.9701	1.9045	4.2763	12.2868	3.6602
Griewank	Mean	0.0306	0.0	0.0	0.0	0.0	0.0	0.0269	0.1537	0.0	0.0
	Std	0.0571	0.0	0.0	0.0	0.0	0.0	0.0453	0.2506	0.0	0.0
	Time	1.3753	2.5003	7.0041	56.6412	36.1668	19.6636	2.3902	0.6494	12.5253	3.7303
Schaffer N.2	Mean	0.0150	0.0004	0.0	0.0	0.0	0.0	0.0095	0.0014	0.0045	0.0007
	Std	0.0201	0.0007	0.0	0.0	0.0	0.0	0.0095	0.0017	0.0067	0.0013
	Time	1.4359	2.6264	7.0888	56.5816	36.7568	20.3590	2.5359	3.9743	12.9016	3.8950
Schaffer N.4	Mean	0.303105	0.292909	0.292587	0.292579	0.292676	0.292665	0.297561	0.293003	0.294915	0.293242
	Std	0.0118	0.0003	0.0	0.0	0.0002	0.0002	0.0040	0.0006	0.0018	0.0007
	Time	1.4997	2.5283	7.1459	58.4871	36.2815	20.2342	2.5560	4.4635	13.2616	4.1806
Schaffer N.6	Mean	0.0418	0.0087	0.0	0.0020	0.0042	0.0071	0.0202	0.0097	0.0100	0.0088
	Std	0.0396	0.0018	0.0	0.0036	0.0048	0.0043	0.0131	0.0003	0.0056	0.0025
	Time	1.4807	2.6265	6.9701	61.2268	35.1733	20.7181	2.6076	4.5070	12.6626	4.0441
Styblinski–Tang	Mean	−38.22368	−39.16615	−39.16617	−39.16617	−39.16614	−39.16617	−37.98762	−39.12722	−39.16617	−39.16617
	Std	3.5263	0.0	0.0	0.0	0.0	0.0	2.2548	0.0699	0.0	0.0
	Time	1.3130	2.4119	6.5984	54.7924	35.2751	19.3258	2.3063	3.9453	12.0077	3.4342
Bukin_6	Mean	1.7159	2.3654	0.1	0.05	2.1321	0.2266	33.6252	6.2734	0.2261	0.2630
	Std	1.1010	1.0687	0.0	0.0001	1.5829	0.1322	20.1480	5.3777	0.2068	0.1325
	Time	0.5719	0.9972	2.8712	21.7811	13.2006	9.9783	0.9815	1.7210	5.7485	1.5428
